# Moxifloxacin-Induced Thrombocytopenia Mediated by Moxifloxacin-Dependent IgM and IgG Antiplatelet Antibodies: A Case Report

**DOI:** 10.7759/cureus.10507

**Published:** 2020-09-17

**Authors:** Joel Moore, Maria R Baer, Brian E Grover, Richard H Aster, Leah S Millstein

**Affiliations:** 1 Departments of Medicine and Pediatrics, University of Maryland School of Medicine, Baltimore, USA; 2 Department of Medicine, University of Maryland School of Medicine, Baltimore, USA; 3 Department of Pharmacy, University of Maryland Medical Center, Baltimore, USA; 4 Department of Medicine/Research, Versiti-Blood Research Institute, Milwaukee, USA; 5 Department of Medicine, Medical College of Wisconsin, Milwaukee, USA

**Keywords:** moxifloxacin, thrombocytopenia, drug-induced, fluoroquinolones

## Abstract

Moxifloxacin is a rare but important cause of drug-induced immune thrombocytopenia (DIT). We describe a patient who presented with an acute onset of severe thrombocytopenia complicated by petechial rash, epistaxis, and melena. Recent new drug exposures included moxifloxacin and two proton pump inhibitors. On presentation to the hospital, all recently initiated medications were discontinued and the patient’s thrombocytopenia was treated with platelet transfusions, intravenous immunoglobulin, and high-dose corticosteroids. Her thrombocytopenia improved over the next seven days and she was discharged on hospital day 8. Serologic testing revealed strongly positive moxifloxacin-dependent IgM and IgG antiplatelet antibodies, confirming a diagnosis of moxifloxacin-induced immune thrombocytopenia. DIT has been reported with other fluoroquinolone antibiotics, especially ciprofloxacin. This case documents a rare but potentially fatal complication of exposure to moxifloxacin and is the first to demonstrate objective evidence of acute sensitization with IgM antibody positivity. It highlights the need to consider this potential reaction when choosing antibiotic therapy, particularly in patients who are at high risk for bleeding, have hematologic disorders, or are receiving myelosuppressive therapies, and perhaps in those with a history of multiple drug allergies.

## Introduction

Thrombocytopenia is common in hospitalized patients and has many possible mechanisms, including decreased platelet production, increased platelet destruction, and splenic sequestration. Medications may cause acute thrombocytopenia, and the pathogenesis of drug-induced immune thrombocytopenia (DIT) can involve complex interactions with the immune system. Heparin-induced thrombocytopenia (HIT), the most common example of DIT, has a unique pathogenesis [1]. Other drugs, including many antibiotics, act by inducing antibodies that bind to platelets only when the sensitizing drug is present; antibody-coated platelets are then destroyed by phagocytic cells of the immune system [2]. Moxifloxacin, a fluoroquinolone antibiotic, is most commonly used to treat respiratory infections, such as bronchitis or community-acquired pneumonia. Serious adverse reactions attributed to the fluoroquinolone class of antibiotics include tendinitis and tendon rupture, dysglycemia, and hepatotoxicity. In 2016, the Food and Drug Administration (FDA) issued a safety alert and recommended against the use of fluoroquinolones as first-line therapy for many infections due to their side effect profile [3]. We present the first report of acute onset of moxifloxacin-induced thrombocytopenia associated with both IgM and IgG antibodies.

## Case presentation

A 55-year-old woman presented to a community hospital emergency department with a diffuse petechial rash, epistaxis, abdominal pain, and melena and was found to have a platelet count of 3,000/mcL. She was hospitalized, transfused two platelet products, and treated with 35 grams of intravenous immunoglobulin (IVIG) and 24 mg IV dexamethasone. She was then transferred to our tertiary care center for further evaluation and management of her thrombocytopenia.

One week prior to the patient’s hospitalization, her primary care provider had prescribed pantoprazole for presumed gastritis. Her platelet count was 322,000/mcL at that time. She developed urticaria the next day, and given a plausible allergic reaction to pantoprazole, she was transitioned to esomeprazole. At a follow-up visit the next day, oral moxifloxacin, 400 mg daily, was started for paronychia.

Past medical history was notable for type 2 diabetes mellitus, hypertension, gastroesophageal reflux disease, and chronic back pain. Chronic medications included metformin, insulin glargine, insulin aspart, losartan, trazodone, tizanidine, and as needed oxycodone/acetaminophen. Previously documented medication allergies included trimethoprim/sulfamethoxazole, ciprofloxacin, and codeine (urticaria), penicillin, amoxicillin, and amoxicillin/clavulanate (rash), and morphine and clindamycin (swelling of the extremities). The patient had reportedly tolerated levofloxacin and moxifloxacin in the past without incident.

**Figure 1:**
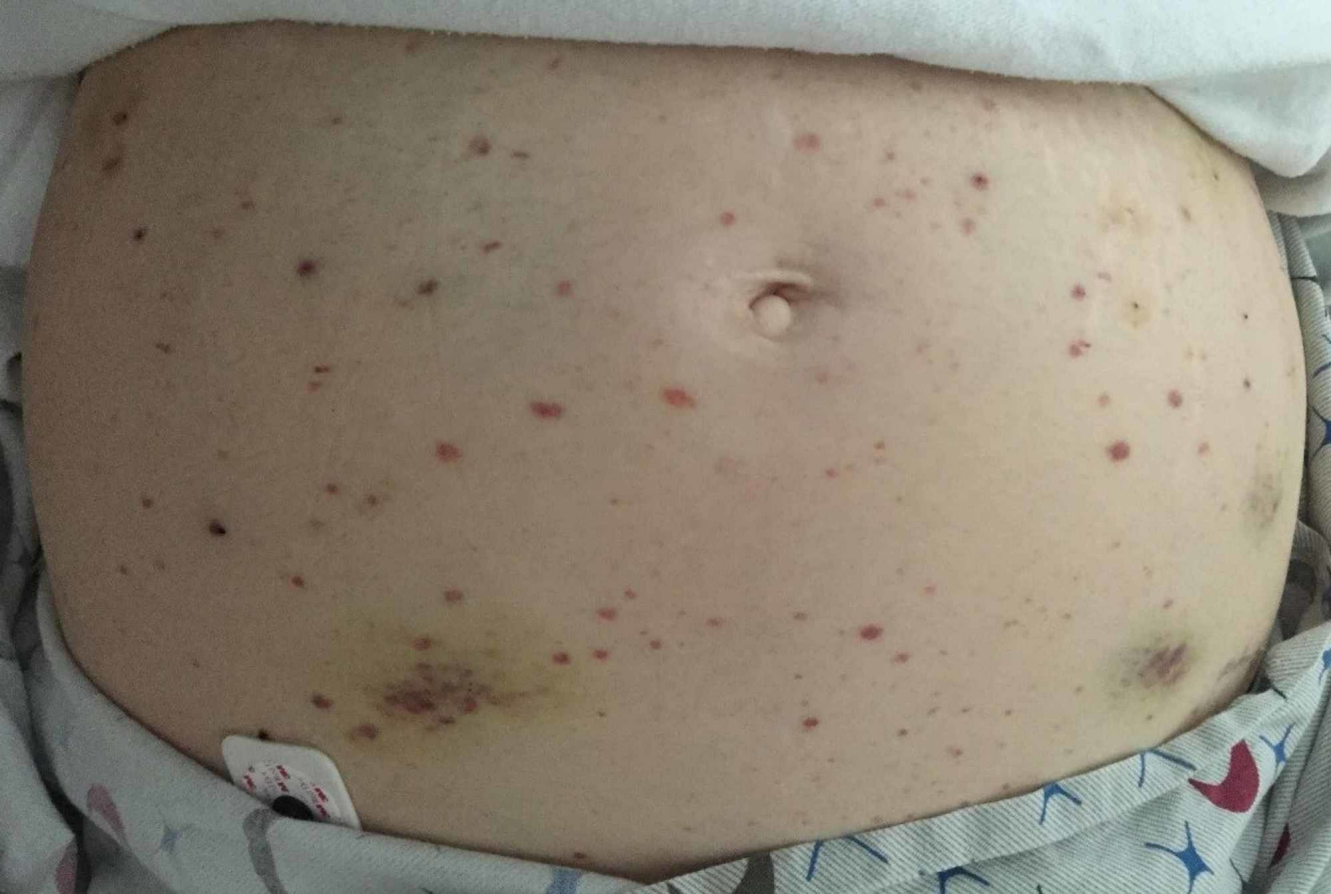
Petechiae and ecchymoses at presentation with severe thrombocytopenia

Upon transfer, she was an anxious-appearing, middle-aged Caucasian woman in no distress, with normal vital signs. She had dried blood on her upper lip, and petechiae and ecchymoses on her left forearm, abdomen (Figure 1), and both legs. The examination was otherwise unremarkable.

On admission to our hospital, hemoglobin (Hgb) was 9.7 g/dL, hematocrit (Hct) was 29.6%, white blood cells (WBC) was 7,900/mcL, and platelet count was 19,000/mcL, following transfusion of two platelet products prior to transfer. Blood chemistries, complete metabolic profile, thyroid-stimulating hormone (TSH), folate level, vitamin B12 level, and coagulation studies were within normal limits, other than hyperglycemia. A stool hemoccult test was positive. 

**Figure 2:**
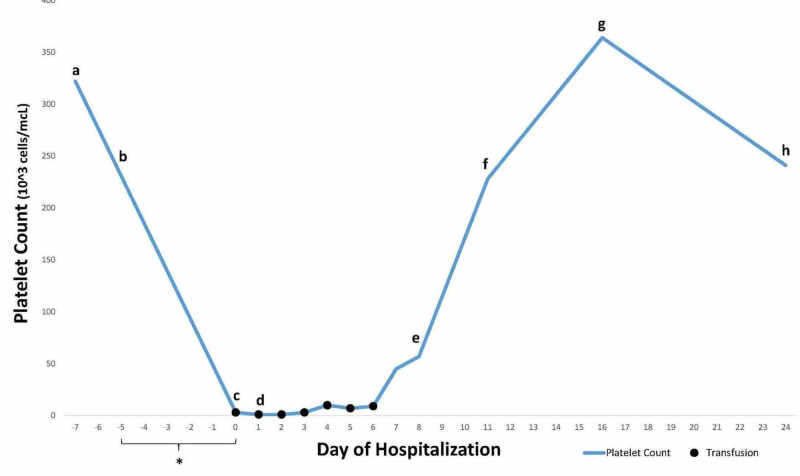
Platelet count over time Day 0 is the day of initial hospitalization. (a) Outside hospital emergency department visit; (b) moxifloxacin initiated; (c) patient hospitalized, moxifloxacin discontinued, (d) patient transferred to tertiary care facility; (e) patient discharged; (f) first follow-up visit, (g) second follow-up visit; (h) third follow-up visit. Black dots indicate platelet transfusions.

The three recently initiated medications were not reinitiated, and the patient was maintained on 1 mg/kg/day IV methylprednisolone. She received nine additional platelet transfusions (11 in total) over the course of eight days to maintain platelet counts above 10,000/mcL (Figure 2). Her epistaxis and melena resolved, and her Hgb and Hct levels remained stable. Her platelet count increased above 50,000/mcL on hospital day 7 and then continued to increase. She was transitioned to an oral prednisone taper and discharged from the hospital. Her platelet count was in the normal range at follow-up visits. Based on the results of her serologic testing (below), she was instructed to avoid future exposure to fluoroquinolone antibiotics.

The patient’s drastic fall in platelet count over the course of one week suggested a diagnosis of DIT in the setting of new drug exposures. On hospital day 4, a whole blood sample was sent to the Blood Center of Wisconsin’s Platelet and Neutrophil Immunology Laboratory for platelet-reactive antibody testing, including moxifloxacin-, esomeprazole-, and pantoprazole-dependent assays.

Serologic testing

Serologic testing was performed using a flow cytometric assay, in which the test serum and normal control serum are incubated with platelets from a normal donor in the presence and absence of the drugs (1.0 mg/ml) implicated as possible causes of the thrombocytopenia. After incubation, the platelets are washed in a buffer containing the same drugs that were present in the primary reaction mixtures, and the platelet-bound antibody is quantified using flow cytometry and fluorescently-labeled IgG- and IgM-specific secondary probes [4]. Using this assay, the patient’s serum was found to contain a strong IgG antibody that recognized platelets in the absence of drug, consistent with an alloantibody or an autoantibody. The strength of the reaction obtained with patient serum increased about 13-fold using an IgG-specific probe and about 2-fold using an IgM-specific probe in the presence of moxifloxacin, but not in the presence of pantoprazole or esomeprazole. The normal serum produced negative reactions in the presence and absence of all three medications.

## Discussion

The time course of exposure to moxifloxacin, the patient’s symptoms, and the presence of IgG and IgM moxifloxacin-dependent, platelet-reactive antibodies indicate that the DIT was caused by a drug-dependent, platelet-reactive antibody induced by moxifloxacin exposure. 

Greinacher et al. divided immune thrombocytopenia induced by drugs into five categories: 1) drug-induced autoimmune thrombocytopenia, 2) drug-dependent immune thrombocytopenia, 3) GP IIb/IIIa antagonist-induced thrombocytopenia, 4) heparin-induced thrombocytopenia (HIT), and 5) drug-induced thrombotic thrombocytopenic purpura (TTP) [5]. Other reports have also detailed multiple mechanisms for this complication of drug treatment [2, 6].

Drug-dependent immune thrombocytopenia differs importantly from drug-induced autoimmune thrombocytopenia. In the former, platelet levels usually recover within a few days of discontinuing the sensitizing medication, whereas, in the latter, autoantibodies induced by the drug may persist and continue to promote platelet destruction, even in the absence of drug [7]. The patient described here had two distinctly different antibodies, one that reacted with normal platelets in the absence of moxifloxacin, and a second, much stronger antibody that required moxifloxacin for binding to platelets. The first antibody was likely an alloantibody induced in the setting of pregnancies or, less likely, an autoantibody, whereas the second antibody was clearly moxifloxacin-dependent.

Fluoroquinolones have rarely been implicated in DIT. A National Center for Biotechnology Information (NCBI) MEDLINE® database search from 2005 - 2020 using the Medical Subject Headings (MeSH®) terms “fluoroquinolones or moxifloxacin” and “thrombocytopenia” revealed 54 results with approximately 20 of them implicating the fluoroquinolone class in the development of thrombocytopenia. Moxifloxacin was implicated in four [8-11]. To our knowledge, only one other case report has documented moxifloxacin-dependent, platelet-reactive antibodies, albeit using a non-specific antibody assay [10]. Researchers have theorized that fluoroquinolone-dependent platelet destruction is due to the structural similarity of fluoroquinolone antibiotics to quinine, which is known to cause platelet-reactive, antibody-mediated immune thrombocytopenia and hemolytic-uremic syndrome (HUS) or TTP [12]. In one proposed model, drugs such as quinine “improve the fit” between complementarity-determining regions of weakly auto-reactive antibodies and target platelet glycoproteins (GPs), such as IIb/IIIa [13-14]. The result is an increased binding affinity between platelet and antibody, thus increasing platelet destruction. It remains unclear whether our patient’s history of multiple drug allergies put her at a higher risk of developing such a complication.

A causal relationship between moxifloxacin exposure and thrombocytopenia is strongly suggested in this case. The probability score on the Naranjo scale was 7, indicating moxifloxacin as the probable cause of thrombocytopenia [15]. Importantly, a normal platelet count was documented prior to exposure, and the platelet count fell by > 99% five days post-moxifloxacin exposure, showing a sustained recovery eight days following discontinuation of the medication. Moreover, serologic testing confirmed the presence of moxifloxacin-dependent IgM and IgG antiplatelet antibodies and showed the absence of esomeprazole- and pantoprazole-dependent antibodies, thus ruling out the only two other new drug exposures as causes. Causation is also supported by prior reports [8-10]. However, causation was not definite in the absence of drug rechallenge, which was not performed because of the potential risks involved.

The time course of drug exposure, development of symptoms, and recovery in our patient followed the typical pattern of DIT, in which exposure to the causative medication is typically five to 10 days prior to the evidence of thrombocytopenia with recovery four to eight days after the causative medication is discontinued [2, 6]. In our patient’s case, the development of severe thrombocytopenia occurred within five days of moxifloxacin exposure, improved seven days after medication discontinuation, and complete recovery was seen at two weeks, consistent with this mechanism. Sensitization occurred in the setting of prior exposure to fluoroquinolones, including ciprofloxacin, which caused an urticarial reaction, and levofloxacin and moxifloxacin which were previously well-tolerated. She was instructed to avoid future exposure to fluoroquinolone antibiotics.

Given the life-threatening thrombocytopenia and bleeding related to thrombocytopenia, the patient was treated with corticosteroids and IV immunoglobulin and received platelet transfusions to attempt to increase her platelet count acutely while awaiting the effect of discontinuation of the sensitizing drug. These interventions were of limited benefit. Platelet transfusions are not contraindicated in immune thrombocytopenia, as they are in TTP, but they are typically minimally effective or ineffective in increasing platelet counts, with or without IV immunoglobulin.

## Conclusions

This case highlights the need for clinicians to be aware of thrombocytopenia as a potential adverse effect of moxifloxacin. It also demonstrates the availability and clinical utility of drug-specific anti-platelet assays, which elucidated the etiology of acute thrombocytopenia in this case and will enable protection of the patient from future exposures to the causative medication. Consideration of moxifloxacin-induced thrombocytopenia is especially important in patients who have preexisting thrombocytopenia, are receiving myelosuppressive medications, or are otherwise at high risk for bleeding. Educating patients on the early warning signs of thrombocytopenia, including petechiae and mucosal bleeding, could be lifesaving.
